# The nonlinear association of ratio of total cholesterol to high density lipoprotein with cognition ability: evidence from a community cohort in China

**DOI:** 10.3389/fnut.2025.1525348

**Published:** 2025-03-05

**Authors:** Yi Zhou, Hao-Ling Xu, Xiao-Ling Lin, Zhi-Ting Chen, Qin-Yong Ye, Zhen-Hua Zhao

**Affiliations:** ^1^Shengli Clinical Medical College of Fujian Medical University, Fuzhou, China; ^2^Department of Neurology, Shengli Clinical Medical College of Fujian Medical University, Fuzhou University Affiliated Provincial Hospital, Fujian Provincial Hospital, Fuzhou, China; ^3^Department of Neurology, Fujian Institute of Geriatrics, Fujian Medical University Union Hospital, Fuzhou, China; ^4^Fujian Key Laboratory of Molecular Neurology, Institute of Clinical Neurology, Institute of Neuroscience, Fujian Medical University, Fuzhou, China; ^5^Department of Neurology, Shengli Clinical Medical College of Fujian Medical University, Fuzhou University Affiliated Provincial Hospital, Fujian Provincial Hospital, Fuzhou, China

**Keywords:** cholesterol, cognition ability, CHARLS, TC-HDL-C ratio, middle-aged, elderly

## Abstract

**Background:**

Cholesterol is a critical component of the human body, particularly within the brain, where it plays an essential role in maintaining cellular structure and function. In addition, the blood lipid profile has been recognized as a significant factor influencing cognitive performance. However, the association between the ratio of total cholesterol (TC) to high-density lipoprotein cholesterol (HDL-C) and cognitive function remains unclear.

**Methods:**

This community-based, cross-sectional study was conducted using data from middle-aged and older adults (aged ≥45 years) participating in the China Health and Retirement Longitudinal Study (CHARLS). The primary exposure of interest was the ratio of TC to HDL-C, while the main outcome was cognitive function, assessed using cognition scores. The association between the TC-HDL-C ratio and cognitive performance was examined using multiple linear regression analyses and restricted cubic spline modeling to assess potential nonlinear relationships.

**Results:**

A total of 8,914 participants were included in the analysis. Within a certain range, a higher TC-HDL-C ratio was significantly associated with improved cognitive scores (*p* < 0.05) among middle-aged and older adults. This relationship remained significant even after adjusting for sociodemographic and health-related factors. Moreover, restricted cubic spline analyses revealed a significant nonlinear association (p for nonlinearity <0.05) between the TC-HDL-C ratio and cognition scores. Subgroup analyses further highlighted differential effects of the TC-HDL-C ratio across specific population subgroups. Sensitivity analyses consistently supported the robustness of the observed relationship between the TC-HDL-C ratio and cognitive function.

**Conclusion:**

Our findings confirm a significant nonlinear relationship between the TC-HDL-C ratio and cognitive performance in middle-aged and elderly individuals, even after adjusting for sociodemographic factors. These results underscore the potential importance of maintaining an appropriate TC-HDL-C ratio to support cognitive health in aging populations.

## 1 Introduction

With the aging population, the prevalence of dementia has risen significantly, presenting major challenges to families and society in China. Dementia is a leading cause of disability and dependency among older adults, with cognitive decline often serving as a precursor to its onset. Therefore, identifying and addressing factors associated with cognitive decline is critical for early prevention and intervention.

Lipid metabolism has been widely studied in relationship to cognitive performance, with conventional lipid parameters such as high-density lipoprotein cholesterol (HDL-C), low-density lipoprotein cholesterol (LDL-C), triglycerides (TG), and total cholesterol (TC) commonly investigated (1–3). In recent years, unconventional lipid parameters, including non-high-density lipoprotein cholesterol (NHDL-C), the NHDL-C/HDL-C ratio, TG/HDL-C ratio, and TC-HDL-C ratio, have been proposed as more sensitive predictors of metabolic and vascular conditions such as diabetes and hypertension (4–7). For example, a retrospective longitudinal study demonstrated that an elevated TG/HDL-C ratio was associated with poorer cognitive performance in individuals with mild cognitive impairment and Alzheimer’s disease (8). However, to date, no studies have examined the relationship between the TC-HDL-C ratio and cognition in Chinese populations.

The TC-HDL-C ratio, which reflects the balance of cholesterol ester-rich lipoproteins, is influenced by both triglyceride and LDL-C levels (9). This parameter has been established as a sensitive predictor of diabetes, as well as cardiovascular and cerebrovascular events, in multiple studies (4, 10, 11). Notably, a long-term prospective study conducted in Sweden identified the TC-HDL-C ratio as a stronger predictor of ischemic heart disease compared to non-HDL-C (12). Furthermore, extreme values of the TC-HDL-C ratio—whether too high or too low—have been associated with increased all-cause mortality in the general population (13). Given the known links between lipid metabolism and diseases such as diabetes, cardiovascular disease, and cognitive dysfunction, exploring the association between the TC-HDL-C ratio and cognition is of significant clinical value.

Despite the growing body of research on lipid parameters, the relationship between the TC-HDL-C ratio and cognitive performance remains unexplored. To address this gap, we conducted a cross-sectional study based on a Chinese community-based cohort. Our study aims to investigate the association between the TC-HDL-C ratio and cognition, providing novel insights into the potential role of lipid metabolism in preventing cognitive decline and supporting public health strategies aimed at reducing the burden of dementia.

## 2 Materials and methods

### 2.1 Study population and ethical statement

The China Health and Retirement Longitudinal Study (CHARLS) is a large-scale interdisciplinary survey implemented jointly by Wuhan University and Peking University. Funded by the National Natural Science Foundation of China (NSFC), CHARLS aims to collect high-quality microdata representing households and individuals aged 45 years and older across China. The study received ethical approval from the Peking University Ethics Committee (Ethics number: IRB00001052–11015), and all participants provided written informed consent (14).

In the 2015 survey, 20,197 participants were enrolled. For the current study, participants were excluded if they were under 45 years old (*N* = 1,010) or had missing data on body mass index (BMI) (*N* = 1,824), gender (*N* = 4), marital status (*N* = 33), hypertension (*N* = 2,074), diabetes (*N* = 170), stroke (*N* = 140), totalcognition scores (*N* = 1,425), the Center for Epidemiological Studies Depression Scale-10 (CESD-10) (*N* = 1,450), drinking status (*N* = 10), education (*N* = 21), sleep duration (*N* = 284), total cholesterol (*N* = 2,238), lipid medications (*N* = 587) or coronary heart disease (*N* = 64). After these exclusions, 8,263 participants were included in the cross-sectional analysis.

### 2.2 Data collection and measurement

Data were collected through face-to-face questionnaire interviews and physical examinations. The questionnaires captured demographic information, self-reported health status, and health-related behaviors. Anthropometric data were measured by trained researchers using standardized protocols (15).

### 2.3 Cognition scores measurement

According to the Health and Retirement Study and CHARLS, two dimensions of cognitive functioning were captured: situational memory and executive functioning. Situational memory is measured by immediate recall and delayed recall (score range, 0–20, with higher scores indicating better functioning). Mental status scores are assessed by orientation, computational, and visuospatial abilities (score range, 0–11, with higher scores indicating better functioning). Global cognition was defined as the total cognition scores of these 2 components with a scale ranging from 0 to 31 points, with higher scores indicating better function (16).

### 2.4 Blood sample collection

Venous blood were collected from primary respondents and their spouses for routine blood tests, including measurements of total cholesterol (TC), high-density lipoprotein cholesterol (HDL-C), ultrasensitive C-reactive protein (hs-CRP), glycated hemoglobin, and genetic markers. Routine blood tests were conducted locally in surveyed counties and cities, while specialized indicators were analyzed in a centralized laboratory designated by the National Center for Disease Control and Prevention (17).

### 2.5 Definition of TC-HDL-C ratio

The formula was used to calculate TC-HDL-C ratio:


$$\begin{array}{l} TC-HDL-C\;\mathrm{ratio}\\ =\mathrm{Total cholesterol}\;\left(\mathrm{mg}/\mathrm{dl}\right)/\mathrm{High density lipoprotein}\;\left(\mathrm{mg}/\mathrm{dl}\right) \end{array}$$

### 2.6 Definitions of covariates

Covariates were selected based on clinical relevance and prior research. Demographic factors included age, gender, residential location, marital status, education level, alcohol consumption, and smoking status. Hypertension and diabetes were identified through self-reports, physical examinations, or routine blood tests. Stroke and coronary heart disease was defined as a participant’s self-report of a physician-diagnosed stroke or coronary heart disease. BMI was calculated as weight (kg) divided by height squared (m^2^). Depression was diagnosed using CESD-10, with scores >9 indicating depression. Sleep duration was calculated as the sum of napping time (hours) and nighttime sleep duration (hours). The usage of lipid medications were defined as a participants self-report.

### 2.7 Statistical analysis

Continuous variables were expressed as mean ± standard deviation (SD) for normally distributed data or median and interquartile range (IQR) for non-normally distributed data. Categorical variables were reported as frequencies and percentages. Between-group comparisons were conducted using the Student’s *t*-test, Chi-square test, or Kruskal-Wallis test.

The association between the TC-HDL-C ratio and cognition scores was analyzed using multiple linear regression. Results were presented as partial regression coefficients (*β*) with 95% confidence intervals (CI). Three models were used for the analysis:

Model I: Unadjusted.

Model II: Adjusted for BMI, age, gender, location, and education.

Model III: Fully adjusted for BMI, age, marital status, gender, location, hypertension, diabetes, stroke, depression, drinking, smoking, education, sleep duration, lipid medications, and coronary heart disease.

Restricted cubic splines (RCS) were employed based on the covariates in Model III to explore potential nonlinear relationships between the TC-HDL-C ratio and cognition scores. Subgroup analyses were also conducted to investigate whether the effect of the TC-HDL-C ratio differed across various populations or conditions. RCS models were applied to the subgroup analyses to identify potential nonlinearities.

### 2.8 Sensitivity analyses

To test the robustness of the results, three sensitivity analyses were performed:

1. Excluding participants with cognition scores greater or less than twice the standard deviation to assess the influence of outliers.
2. Employing robust linear regression to account for the effects of extreme values.
3. Using a generalized additive model (GAM) to confirm the nonlinear association between the TC-HDL-C ratio and cognition scores.

All statistical analyses were performed using R software (version 4.1.3) and EmpowerStats (version 4.2). A *p*-value <0.05 was considered statistically significant.

## 3 Results

### 3.1 Baseline characteristics of participants based on TC-HDL-C ratio quartiles

A total of 8,914 participants met the inclusion criteria and were enrolled in the analysis. Participants were divided into four groups based on the quartiles of the TC-HDL-C ratio: Q1 (≤ 3.089), Q2 (3.089 to ≤3.63), Q3 (3.63 to ≤4.24), and Q4 (≥ 4.24).

No significant differences were observed in BMI and age across the four groups. Similarly, gender, marital status, diabetes status, and sleep duration were not significantly different among the groups. However, higher TC-HDL-C ratios were associated with increased prevalence of stroke, hypertension, alcohol consumption, and smoking. Participants in the higher TC-HDL-C ratio quartiles were more likely to live in rural areas and have lower levels of education (Table 1).

**Table 1:** Baseline characteristics of the selected participants.

Variables	Total (*n* = 8,326)	1 (*n* = 2,082)	2 (*n* = 2,081)	3 (*n* = 2,081)	4 (*n* = 2,082)	Statistic	*P*
Sleep duration, M (Q_1_, Q_3_)	6.00 (5.00, 8.00)	6.00 (5.00,8.00)	6.00 (5.00,8.00)	6.00 (5.00,8.00)	7.00 (5.00,8.00)	χ^2^ = 22.70#	**<0.001**
Age, M (Q_1_, Q_3_)	61.00 (53.00, 67.00)	61.00 (53.00,67.00)	60.00 (53.00,66.00)	60.00 (53.00,67.00)	61.00 (54.00,67.00)	χ^2^ = 7.32#	0.062
BMI, M (Q_1_, Q_3_)	23.74 (21.38, 26.30)	21.76 (19.89,23.96)	23.51 (21.25,25.77)	24.45 (22.38,26.73)	25.13 (23.01,27.61)	χ^2^ = 994.54#	**<0.001**
Episodic memory scores, M (Q_1_, Q_3_)	7.00 (5.00, 9.00)	7.00 (4.00,9.00)	7.00 (5.00,10.00)	7.00 (5.00,10.00)	7.00 (5.00,10.00)	χ^2^ = 42.67#	**<0.001**
Mental status scores, M (Q_1_, Q_3_)	9.00 (7.00, 10.00)	9.00 (7.00,10.00)	9.00 (7.00,10.00)	9.00 (7.00,10.00)	9.00 (7.00,10.00)	χ^2^ = 11.59#	**0.009**
Total cognition scores, M (Q_1_, Q_3_)	12.50 (9.50, 14.50)	12.00 (9.00,14.00)	12.50 (9.50,14.50)	12.50 (10.00,14.50)	12.50 (9.50,14.50)	χ^2^ = 28.20#	**<0.001**
Coronary heart disease, n(%)						χ^2^ = 46.80	**<0.001**
No	6,769 (81.93)	1764 (85.34)	1726 (83.58)	1,677 (81.17)	1,602 (77.62)		
Yes	1,493 (18.07)	303 (14.66)	339 (16.42)	389 (18.83)	462 (22.38)		
Lipid medication, n(%)						χ^2^ = 100.84	**<0.001**
No	7,677 (92.21)	1988 (95.49)	1960 (94.19)	1901 (91.35)	1828 (87.80)		
Yes	649 (7.79)	94 (4.51)	121 (5.81)	180 (8.65)	254 (12.20)		
Gender, n(%)						χ^2^ = 43.52	**<0.001**
Female	4,144 (49.77)	907 (43.56)	1,083 (52.04)	1,090 (52.38)	1,064 (51.10)		
Male	4,182 (50.23)	1,175 (56.44)	998 (47.96)	991 (47.62)	1,018 (48.90)		
Marriage status, n(%)						χ^2^ = 4.43	0.219
No	963 (11.57)	259 (12.44)	252 (12.11)	221 (10.62)	231 (11.10)		
Yes	7,363 (88.43)	1823 (87.56)	1829 (87.89)	1860 (89.38)	1851 (88.90)		
Rural, n(%)						χ^2^ = 116.92	**<0.001**
No	3,208 (38.53)	655 (31.46)	728 (34.98)	857 (41.18)	968 (46.49)		
Yes	5,118 (61.47)	1,427 (68.54)	1,353 (65.02)	1,224 (58.82)	1,114 (53.51)		
Depression, n(%)						χ^2^ = 4.53	0.210
No	5,642 (67.76)	1,374 (65.99)	1,415 (68.00)	1,417 (68.09)	1,436 (68.97)		
Yes	2,684 (32.24)	708 (34.01)	666 (32.00)	664 (31.91)	646 (31.03)		
Education, n(%)						χ^2^ = 41.63	**<0.001**
Primary school below	3,171 (38.09)	856 (41.11)	823 (39.55)	777 (37.34)	715 (34.34)		
Primary school	2066 (24.81)	540 (25.94)	515 (24.75)	497 (23.88)	514 (24.69)		
Middle school	2047 (24.59)	471 (22.62)	505 (24.27)	521 (25.04)	550 (26.42)		
High school and above	1,042 (12.52)	215 (10.33)	238 (11.44)	286 (13.74)	303 (14.55)		
Hypertension, n(%)						χ^2^ = 111.41	**<0.001**
No	4,117 (49.45)	1,184 (56.87)	1,089 (52.33)	983 (47.24)	861 (41.35)		
Yes	4,209 (50.55)	898 (43.13)	992 (47.67)	1,098 (52.76)	1,221 (58.65)		
Diabetes, n(%)						χ^2^ = 230.62	**<0.001**
No	6,779 (81.42)	1836 (88.18)	1797 (86.35)	1,652 (79.38)	1,494 (71.76)		
Yes	1,547 (18.58)	246 (11.82)	284 (13.65)	429 (20.62)	588 (28.24)		
Smoking, n(%)						χ^2^ = 55.07	**<0.001**
No	4,486 (53.88)	993 (47.69)	1,180 (56.70)	1,211 (58.19)	1,102 (52.93)		
Yes	3,840 (46.12)	1,089 (52.31)	901 (43.30)	870 (41.81)	980 (47.07)		
Drinking, n(%)						χ^2^ = 74.04	**<0.001**
No	4,322 (51.91)	913 (43.85)	1,113 (53.48)	1,139 (54.73)	1,157 (55.57)		
Yes	4,004 (48.09)	1,169 (56.15)	968 (46.52)	942 (45.27)	925 (44.43)		

Q1–Q4 was divided according to the quartile of TC-HDL-C ratio; #: Kruskal-waills test, χ^2^: Chi square test. Bold values mean statistical significance.

### 3.2 Association between TC-HDL-C ratio and cognitive ability among middle-aged and elder population

In the unadjusted model, multiple linear regression analysis revealed a positive association between TC-HDL-C ratio and cognitive scores (p for trend <0.001) (Table 2). To confirmed the analysis, partially adjusted and fully adjusted models were employed. In Model II, participants with TC-HDL-C ratio between 3.089 and 4.24 (Q2 and Q3) demonstrated better cognitive scores compared to those in Q1 (≤ 3.089) (Q2 compared to Q1: *β*: 0.40, *p* = 0.003; Q3 compared to Q1: *β*: 0.34, *p* = 0.011) (Table 2).

**Table 2:** Multiple linear regression between TC-HDL-C ratio and total cognition scores.

Variables	Model1		Model2		Model3	
	β (95%CI)	*P*	β (95%CI)	*P*	β (95%CI)	*P*
TC/HDL-C quantile						
Q1	0.00 (Reference)		0.00 (Reference)		0.00 (Reference)	
Q2	0.64 (0.33 ~ 0.94)	**<0.001**	0.40 (0.14 ~ 0.66)	**0.003**	0.38 (0.12 ~ 0.63)	**0.004**
Q3	0.76 (0.46 ~ 1.07)	**<0.001**	0.34 (0.08 ~ 0.60)	**0.011**	0.34 (0.08 ~ 0.59)	**0.011**
Q4	0.84 (0.53 ~ 1.14)	**<0.001**	0.23 (−0.04 ~ 0.49)	0.092	0.25 (−0.01 ~ 0.52)	0.063
p for trend	**<0.001**		0.151		0.097	

CI, Confidence Interval. Model1: Crude. Model2: Adjust: gender, rural, age, education, BMI. Model3: Adjust: gender, marriage status, location, depression, sleep duration, age, education, hypertension, diabetes, BMI, smoking, drinking, stroke, coronary heart disease, lipid medication. Bold values mean statistical significance.

Interestingly, no protective effect of TC/HDL-C was observed for participants in Q4 (≥ 4.26) (*β*: 0.25, *p* = 0.063) (Table 2). The results of the fully adjusted Model III were consistent with those of Model II (Q2 compared to Q1: β: 0.38, *p* = 0.004; Q3 compared to Q1: β: 0.34, *p* = 0.011) (Table 2).

Given that cognition scores in the CHARLS survey comprise episodic memory scores and mental status scores, the association between TC-HDL-C ratio and these components was further investigated. Interestingly, in fully adjusted model III, TC-HDL-C ratio was positively correlated with episodic memory scores (Q2 compared to Q1: *β*: 0.32, *p* < 0.001; Q3 compared to Q1: β: 0.29, *p* = 0.003; Q4 compared to Q1: β: 0.32, *p* = 0.001) (Supplementary Table S1). However, no linear relationship was identified between TC-HDL-C ratio and mental status scores (Q2 compared to Q1: β: 0.06, *p* = 0.38; Q3 compared to Q1: β: 0.04, *p* = 0.574; Q4 compared to Q1: β: −0.07, *p* = 0.334) (Supplementary Table S2).

### 3.3 Nonlinear association between TC-HDL-C ratio and cognitive ability

**Figure 1:**
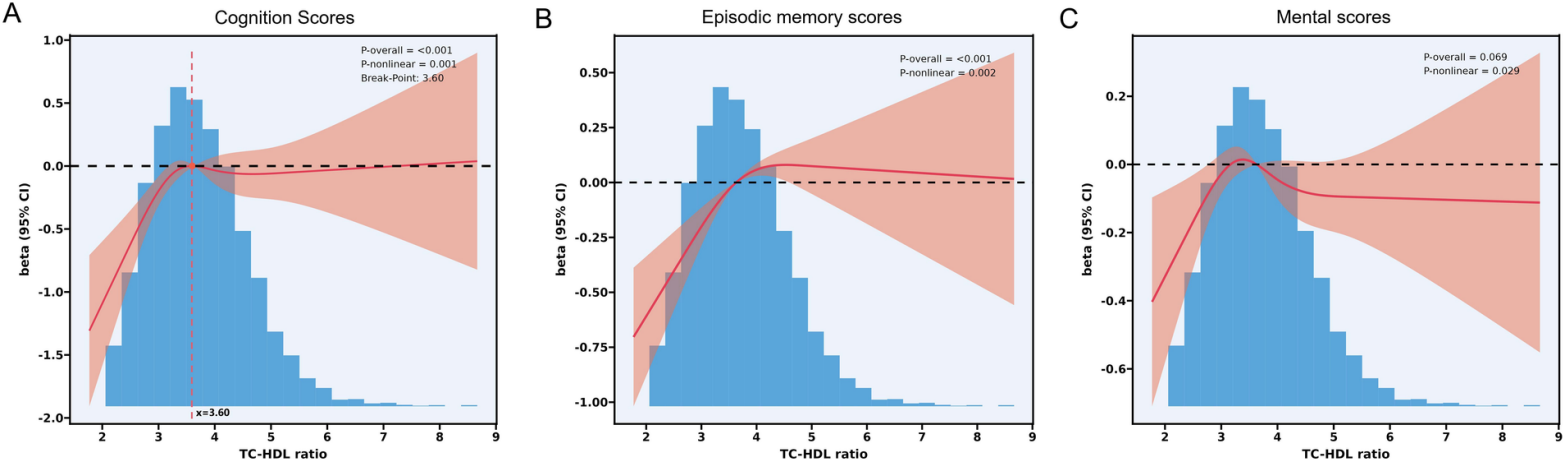
**(A)** RCS analysis between TC-HDL-C ratio and total cognition scores. p for overall <0.001; p for nonlinear = 0.001. Breaking point = 3.60. **(B)** RCS analysis between TC-HDL-C ratio and mental status scores. p for overall = 0.069; p for nonlinear = 0.029. **(C)** RCS analysis between TC-HDL-C ratio and episodic memory scores. p for overall <0.001; p for nonlinear = 0.002.

To further explore the relationship between TC-HDL-C ratio and cognitive ability, restricted cubic spline (RCS) analysis was performed. The results indicated that a nonlinear model provided a better fit than a linear model after adjusting for all covariates (p for overall <0.001, p for nonlinear = 0.001, turning point = 3.60) (Figure 1A). Specifically, cognition scores increased with TC-HDL-C ratio up to a turning point (TC-HDL-C ratio = 3.60). Beyond this threshold, total cognition scores declined slightly and then plateaued.

Piecewise linear regression analysis revealed a positive association between TC-HDL-C ratio and cognition scores when TC/HDL-C was below the turning point (Model I: *β* = 0.86, *p* < 0.001; Model II: *β* = 0.58, *p* < 0.001; Model III: *β* = 0.55, *p* < 0.001) (TC-HDL-C ratio = 3.60) (Table 3). However, no significant linear relationship was observed beyond this threshold. (Model I: *β* = 0.07, *p* < 0.416; Model II: *β* = −0.08, *p* = 0.295; Model III: *β* = −0.05, *p* < 535) (Table 3).

**Table 3:** Piecewise multiple linear regression between TC-HDL-C ratio and total cognition scores according to turning point.

Variables	Model1			Model2			Model3	
	β (95%CI)	*P*		β (95%CI)	*P*		β (95%CI)	*P*
TC/HDL-C ≤ 3.60	0.86 (0.57 ~ 1.15)	<0.001		0.58 (0.20 ~ 0.91)	<0.001		0.55 (0.30 ~ 0.81)	<0.001
TC/HDL-C > 3.60	0.07 (−0.10 ~ 0.24)	0.416		−0.08 (−0.23 ~ 0.07)	0.295		−0.05 (−0.20 ~ 0.10)	0.535

CI, Confidence Interval. Model1: Crude. Model2: Adjust: gender, rural, age, education, BMI. Model3: Adjust: gender, marriage status, rural, depression, sleep duration, age, education, hypertension, diabetes, BMI, smoking, drinking, stroke, coronary heart disease, lipid medication.

RCS analysis also detected nonlinear relationships between TC-HDL-C ratio and both episodic memory scores and mental status scores (Figures 1B,C). A strong nonlinear association was observed between TC-HDL-C ratio and episodic memory scores (p for overall = 0.069; p for nonlinear = 0.029) (Figure 1B). Similarly, a nonlinear relationship was detected between TC-HDL-C ratio and mental status score (p for overall = 0.069, p for nonlinearity = 0.029) (Figure 1C).

### 3.4 Subgroup analysis

**Figure 2:**
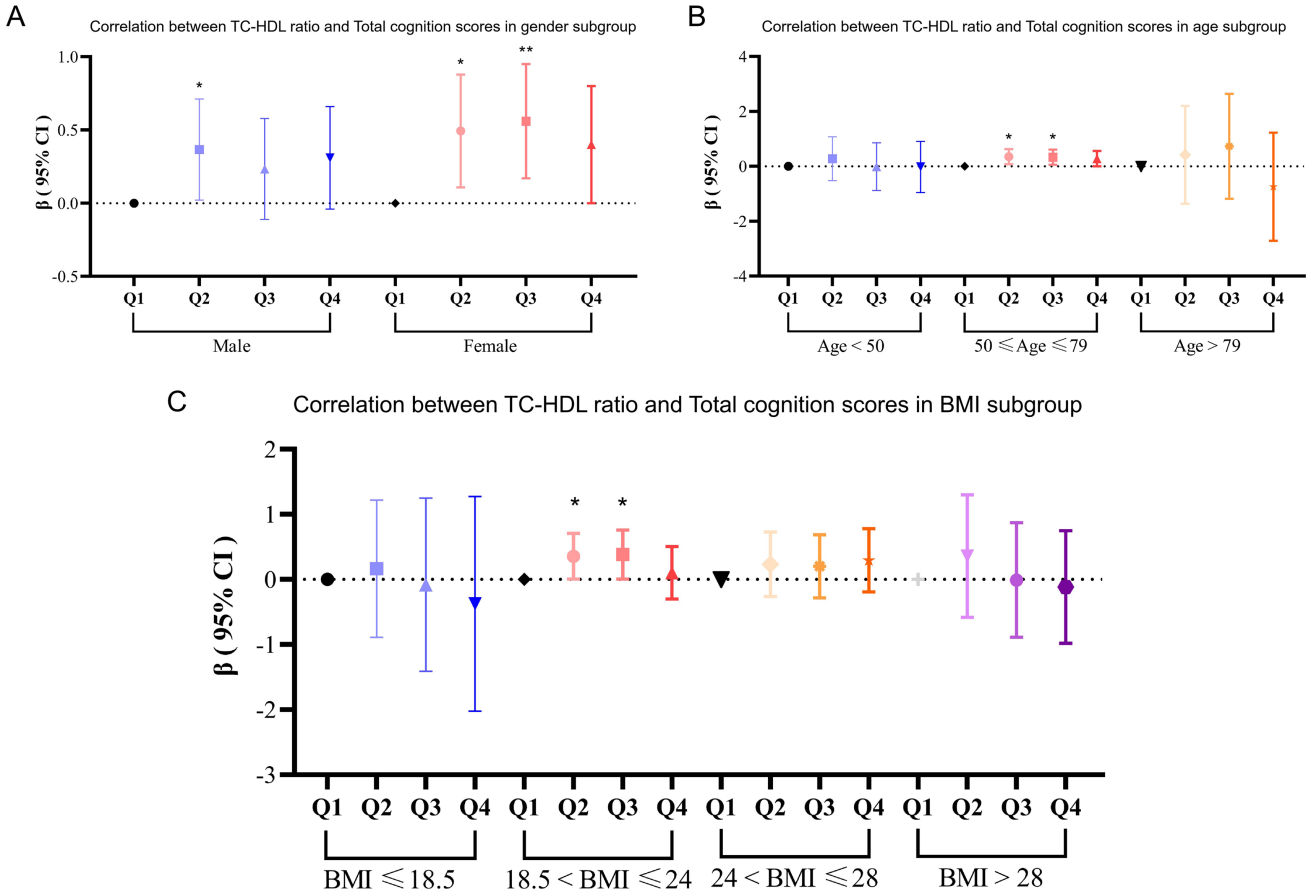
**(A)** Subgroup analysis between TC-HDL-C ratio and total cognition scores according to age. **(B)** Subgroup analysis between TC-HDL-C ratio and total cognition scores according to gender. **(C)** Subgroup analysis between TC-HDL-C ratio and total cognition scores according to BMI.

Subgroup analyses revealed that the association between TC-HDL-C ratio and cognitive scores varied by gender. Among females, participants in Q2 and Q3 had significantly higher cognitive scores compared to those in Q1 (Q2 compared to Q1: *β* = 0.48, *p* = 0.014; Q3 compared to Q1: *β* = 0.51, *p* = 0.010). Meanwhile, participants in Q2 had significantly higher total cognition scores compared to those in Q1 (*β* = 0.34, *p* = 0.048) (Figure 2A). In age subgroup, participants of Q2 and Q3 groups that aged between 50 years to 79 years had a significant relationship between TC-HDL-C ratio and total cognition scores (Q2 compared to Q1: *β* = 0.38, *p* = 0.007; Q3 compared to Q1: *β* = 0.35, *p* = 0.012) (Figure 2B). For BMI subgroup analysis, multiple linear regression shows that, in BMI between 18.5 and 24 subgroup, participants in Q3 and Q2 had a strong linear relationship between TC-HDL-C ratio and cognition scores (Q2 compared to Q1: *β* = 0.35, *p* = 0.048; Q3 compared to Q1: *β* = 0.38, *p* = 0.047) (Figure 2C).

**Figure 3:**
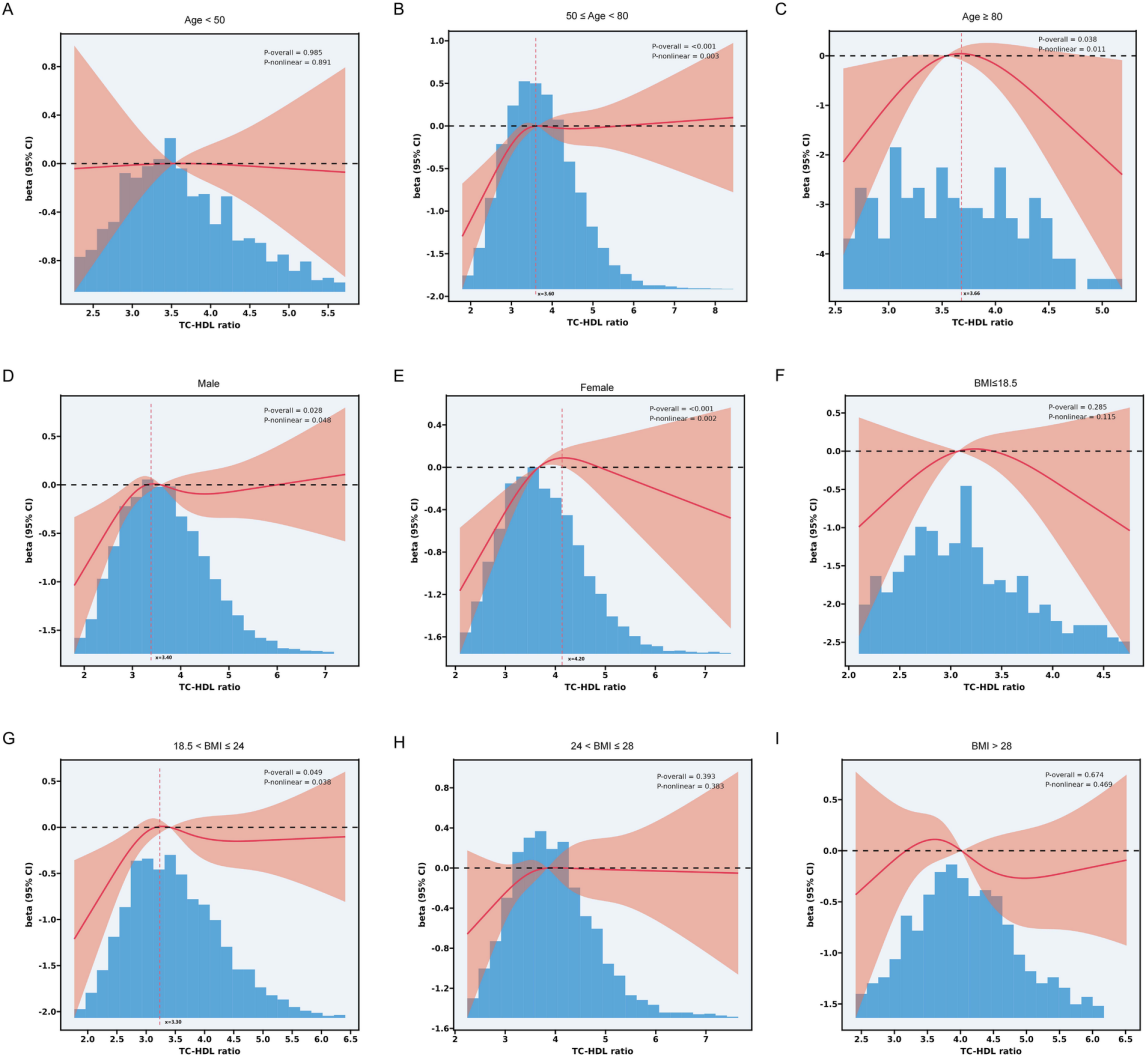
**(A)** Subgroup RCS analysis between TC-HDL-C ratio and total cognition scores in participants aged < 50 years. **(B)** Subgroup RCS analysis between TC-HDL-C ratio and total cognition scores in participants aged between 50-79 years. **(C)** Subgroup RCS analysis between TC-HDL-C ratio and total cognition scores in participants aged ≥ 80. **(D)** Subgroup RCS analysis between TC-HDL-C ratio and total cognition scores in male. **(E)** Subgroup RCS analysis between TC-HDL-C ratio and total cognition scores in female. **(F)** Subgroup RCS analysis between TC-HDL-C ratio and total cognition scores in participants whose BMI ≤ 18.5. **(G)** Subgroup RCS analysis between TC-HDL-C ratio and total cognition scores in participants whose BMI between 18.5 and 24. **(H)** Subgroup RCS analysis between TC-HDL-C ratio and total cognition scores in participants whose BMI between 24 and 28. **(I)** Subgroup RCS analysis between TC-HDL-C ratio and total cognition scores in participants whose BMI > 28.

Meanwhile, RCS analysis demonstrated significant nonlinear relationships within each subgroup. For participants aged between 50 years and 79 years, the relationship between TC-HDL-C ratio and total cognition scores followed an inverse L-shaped curve (p for nonlinear = 0.003, turning points = 3.60). In contrast, for participants aged ≥80 years, an inverse U-shaped curve was found (p for nonlinear = 0.011, turning points = 3.66) (Figures 3A–C). In male subgroup, TC-HDL-C ratio had an nearly inverse L-shaped curve with total cognition scores. In addition, in female, TC-HDL-C ratio had an inverse U-shaped curve with total cognition ability, which suggests that too-high and too-low TC-HDL-C ratio might increase the risk of cognition decline (Figures 3D,E). However, in BMI subgroup, only participants whose BMI between 18.5 and 24 had a nonlinear association between TC-HDL-C ratio and total cognition scores (p for nonlinear = 0.038) (Figures 3F–I).

### 3.5 Sensitivity analyses

To ensure the robustness of the findings, a series of sensitivity analyses was conducted:

1. Participants with cognition scores greater or less than twice the standard deviation were excluded. The results showed that the Q2 group remained linearly associated with cognition scores (Supplementary Table S3).
2. Robust regression confirmed the main findings (Q2 compared to Q1: *β* = 0.037, *p* = 0.013) (Supplementary Table S4).
3. Generalized additive models (GAM) supported the nonlinear relationships identified in the RCS analysis, showing similar trends (p for smooth term = 0.0303) (Supplementary Figure 1).

## 4 Discussion

The relationship between blood lipid levels and cognition is complex and has yielded conflicting findings across studies. While some research suggests that higher total cholesterol (TC) increases the risk of mild cognitive impairment (1, 18, 19), a double-blind, randomized, placebo-controlled trial reported that lowering lipid levels does not confer protective effects on cognition in older adults (20). Conversely, other studies have found that elevated serum total cholesterol is associated with better cognitive performance (21, 22). Recent evidence indicates that the relationship between plasma lipid levels and cognition may be nonlinear (3, 23, 24). Although high-density lipoprotein cholesterol (HDL-C) has traditionally been viewed as protective for cognitive health, emerging research suggests that excessive HDL-C levels might impair cognition (25–27). These discrepancies can largely be attributed to heterogeneity in study populations, variations in methods of assessing cognitive function, and differences in study designs. Together, these findings suggests the complexity of the relationship between lipid metabolism and cognition, highlighting it as a challenging topic for research.

Unconventional lipid parameters have gained attention for their potential to elucidate the relationship between lipid metabolism and cognition. High remnant cholesterol (RC) has been associated with poor cognitive outcomes, and lower RC/TC ratios may reduce the risk of cognitive impairment. In China, a cross-sectional study reported a U-shaped relationship between non-HDL-C levels and cognitive scores (28). However, longitudinal research has suggested no significant association between the TC-HDL-C ratio and dementia development (29). To our knowledge, the present study is the first to specifically investigate the association between the TC-HDL-C ratio and cognitive function in a Chinese population. Given the widespread use of the TC-HDL-C ratio as a predictor of metabolic disorders such as atherosclerosis, cardiovascular disease, and diabetes (12, 30), Its potential link to cognitive function is of interest. Notably, individuals with both Alzheimer’s disease and metabolic disorders exhibit worse cognitive performance than those without metabolic disorders (31). Additionally, diabetes has been shown to be associated with mild cognitive dysfunction, further supporting the connection between metabolic health and cognition (32). However, studies on the TC-HDL-C ratio as a lipid metabolic parameter in relation to cognitive function remain scarce.

Mechanistically, cholesterol plays a vital role in neuronal function. As an essential component of cell membranes, cholesterol is particularly critical in the central nervous system, where it is synthesized by astrocytes and transported to neurons. It is indispensable for processes such as myelination, synaptic plasticity, and neuronal signaling, all of which underpin cognitive function. Cholesterol deficiency in animal models has been shown to impair learning and memory, effects that can be reversed by cholesterol supplementation (33, 34). In aging and disease models, such as Huntington’s disease, increasing brain cholesterol levels has been linked to improved synaptic function and cognitive performance (35, 36). These findings suggest that higher TC levels may have protective effects on cognition, though this relationship is likely context-dependent and influenced by other metabolic factors.

Traditional views have categorized LDL as detrimental and HDL-C as beneficial, but emerging evidence challenges this binary perspective (37). High HDL-C levels, for instance, have been associated with increased fracture risk in older adults (38), and non-HDL-C levels have been positively correlated with global cognitive function (24). These findings prompt a reassessment of the relationships between different lipid fractions and cognition.

Our study, based on a large Chinese cohort, is the first to uncover a nonlinear relationship between the TC-HDL-C ratio and cognitive scores, identifying a turning point at a ratio of 3.50. Interestingly, the effects of the TC-HDL-C ratio differed by age group. Among middle-aged participants, cognitive performance plateaued at the turning point. In contrast, among older adults, cognitive performance worsened with increasing TC-HDL-C ratios beyond the turning point. This dose–response relationship was consistent across gender subgroup analyses, further supporting the robustness of our findings.

This study has several strengths. First, it is based on a large, community-based cohort of middle-aged and older adults, enhancing the generalizability of the findings to similar populations. Second, the use of advanced statistical methods, such as restricted cubic splines and sensitivity analyses, allowed us to robustly examine the nonlinear relationships between TC-HDL-C ratio and cognition.

However, several limitations should be acknowledged. As a cross-sectional study, we were unable to infer causality between the TC-HDL-C ratio and cognitive performance. Although we adjusted for numerous potential confounders, residual confounding remains a possibility. Many studies have revealed that changes in hormone levels will lead to changes in lipid metabolism profiles. Thyroid hormones affect lipid metabolism by regulating lipid synthesis and catabolism in the liver. Hypothyroidism usually leads to higher cholesterol levels, while hyperthyroidism may lead to lower cholesterol levels. In addition to this, thyroid hormones have the ability to affect cognition directly by influencing brain function. Thus, the lack of data on thyroid-related disorders would lead to a possible bias in our findings. Numerous studies has demonstrated that the menopause is associated with significant hormonal changes that can affect both lipid levels and cognitive performance (39). In this study, due to the lack of data on menopause, this leads us to conclude that there may be a potential bias among women with perimenopausal syndrome. Additionally, the study population was ethnically homogenous, limiting the generalizability of our findings to diverse populations. For people of different age groups, their cognitive ability thresholds are age-specific. Due to the lack of recognized thresholds for the cognitive scores used by CHARLS in the Chinese population, this may lead to either an overestimation or underestimation of the relationship between the TC-HDL-C ratio and cognition. Future longitudinal studies are needed to validate these findings and clarify the causal relationship between the TC-HDL-C ratio and cognitive outcomes. Research on more diverse populations is also necessary to enhance the applicability of these results to global populations.

## 5 Conclusion

Our study provides novel insights into the relationship between the TC-HDL-C ratio and cognitive function. We identified a significant nonlinear association, with a turning point at a TC-HDL-C ratio of 3.60. These findings emphasize the need to consider lipid metabolism in cognitive health assessments and suggest that maintaining an optimal TC-HDL-C ratio may help preserve cognitive function, particularly in aging populations. Further longitudinal and multicenter studies are warranted to confirm these findings and explore their broader implications for public health and clinical practice.

## Data Availability

The original contributions presented in the study are included in the article/Supplementary material, further inquiries can be directed to the corresponding authors.
