# Supplementary material for: The nonlinear association of ratio of total cholesterol to high density lipoprotein with cognition ability: evidence from a community cohort in China
Source: Front Nutr. 2025 Mar 5;12:1525348. doi: 10.3389/fnut.2025.1525348 (PMC11919651; doi:10.3389/fnut.2025.1525348)
Supplement: Supplementary file 1 [file Data_Sheet_1.docx]

Supplementary Material

# Supplementary Figures and Tables

For more information on Supplementary Material and for details on the different file types accepted, please see [here](https://www.frontiersin.org/guidelines/author-guidelines#supplementary-material).

## Supplementary Figures


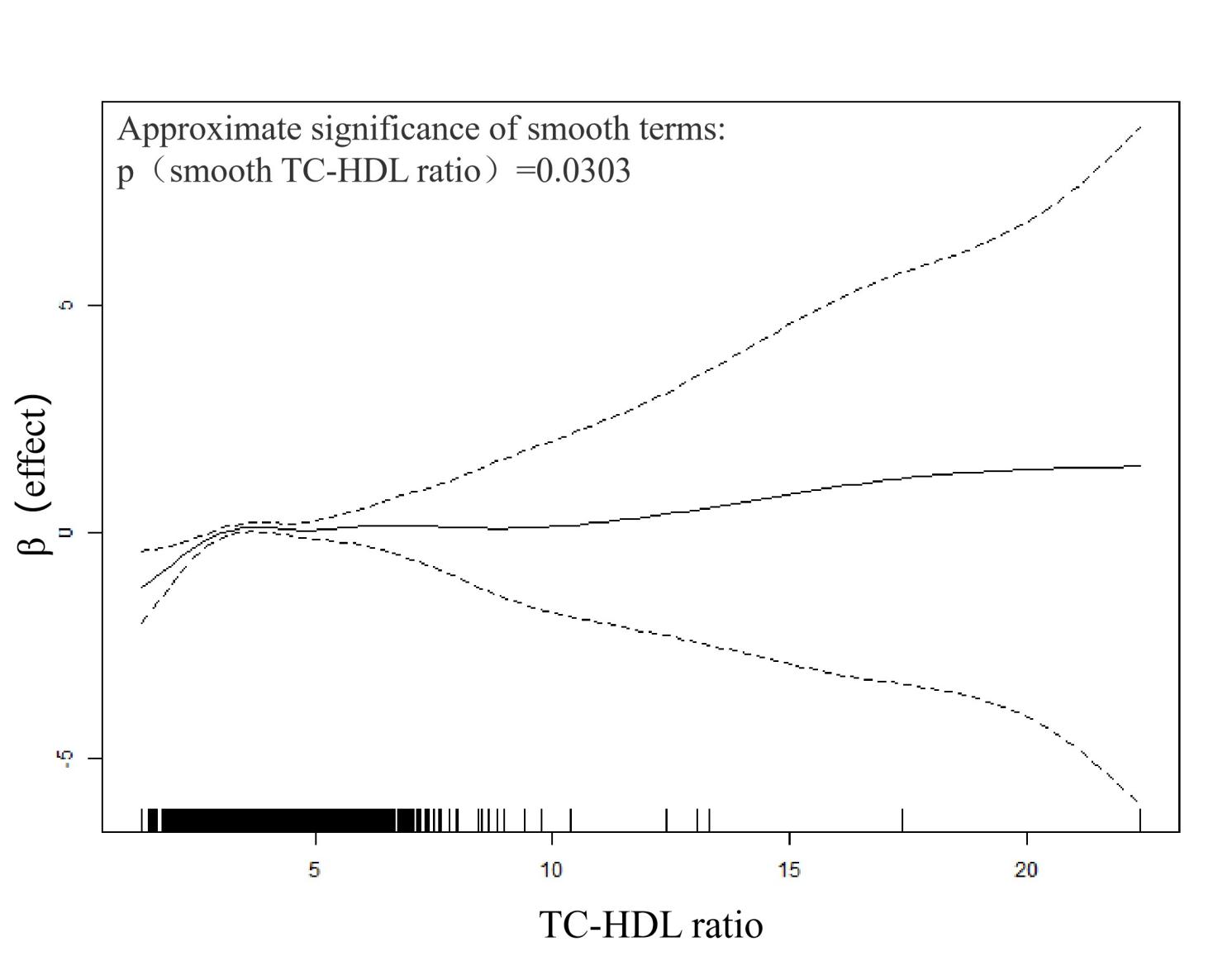


**Supplementary Figure 1.** GAM analysis of TC-HDL ratio and cognition score, Adjust: gender, marriage status, rural, Depression, sleep, age, education, hypertension, diabetes, BMI, smoking, drinking, stroke, lipid medications, coronary heart diseases. p (smooth term TC-HDL ratio) = 0.0303.

## Supplementary Table

### Table.S1 Multiple linear regression between TC-HDL-C ratio and episodic memory scores

| Variables | Model1 | |  | Model2 | |  | Model3 | |
| --- | --- | --- | --- | --- | --- | --- | --- | --- |
|  | β (95%CI) | *P* |  | β (95%CI) | *P* |  | β (95%CI) | *P* |
| TC/HDL quantile |  |  |  |  |  |  |  |  |
| 1 | 0.00 (Reference) |  |  | 0.00 (Reference) |  |  | 0.00 (Reference) |  |
| 2 | 0.12 (-0.03 ~ 0.26) | 0.114 |  | 0.33 (0.14 ~ 0.52) | **<.001** |  | 0.32 (0.13 ~ 0.51) | **<.001** |
| 3 | 0.21 (0.07 ~ 0.36) | **0.004** |  | 0.29 (0.09 ~ 0.48) | **0.004** |  | 0.29 (0.10 ~ 0.48) | **0.003** |
| 4 | 0.20 (0.06 ~ 0.35) | **0.007** |  | 0.30 (0.10 ~ 0.49) | **0.003** |  | 0.33 (0.13 ~ 0.52) | **0.001** |
| CI: Confidence Interval | | | | | | | | |
| Model1: Crude | | | | | | | | |
| Model2: Adjust: gender, rural, age, education, BMI | | | | | | | | |
| Model3: Adjust: gender, marriage status, rural, Depression, sleep, age, education, hypertension, diabetes, BMI, smoking, drinking, stroke, lipid medications, coronary heart diseases | | | | | | | | |
|  | | | | | | | | |

**Table.S2** Multiple linear regression between TC-HDL-C ratio and mental status scores

| Variables | Model1 | |  | Model2 | |  | Model3 | |
| --- | --- | --- | --- | --- | --- | --- | --- | --- |
|  | β (95%CI) | *P* |  | β (95%CI) | *P* |  | β (95%CI) | *P* |
| TC/HDL quantile |  |  |  |  |  |  |  |  |
| 1 | 0.00 (Reference) |  |  | 0.00 (Reference) |  |  | 0.00 (Reference) |  |
| 2 | 0.12 (-0.03 ~ 0.27) | 0.126 |  | 0.07 (-0.06 ~ 0.20) | 0.292 |  | 0.06 (-0.07 ~ 0.19) | 0.380 |
| 3 | 0.20 (0.04 ~ 0.35) | **0.011** |  | 0.05 (-0.08 ~ 0.18) | 0.482 |  | 0.04 (-0.09 ~ 0.17) | 0.574 |
| 4 | 0.20 (0.05 ~ 0.35) | **0.011** |  | -0.06 (-0.20 ~ 0.07) | 0.349 |  | -0.07 (-0.20 ~ 0.07) | 0.344 |
| CI: Confidence Interval | | | | | | | | |
| Model1: Crude | | | | | | | | |
| Model2: Adjust: gender, rural, age, education, BMI | | | | | | | | |
| Model3: Adjust: gender, marriage status, rural, Depression, sleep, age, education, hypertension, diabetes, BMI, smoking, drinking, stroke, lipid medications, coronary heart diseases | | | | | | | | |

**Table.S3** Multiple linear regression between TC-HDL-C ratio and cognition scores, after excluding those with cognitive scores outside of two standard deviations

| Variables | Model1 | |  | Model2 | |  | Model3 | |
| --- | --- | --- | --- | --- | --- | --- | --- | --- |
|  | β (95%CI) | *P* |  | β (95%CI) | *P* |  | β (95%CI) | *P* |
| TC/HDL quantile |  |  |  |  |  |  |  |  |
| 1 | 0.00 (Reference) |  |  | 0.00 (Reference) |  |  | 0.00 (Reference) |  |
| 2 | 0.45 (0.17 ~ 0.72) | **0.002** |  | 0.28 (0.04 ~ 0.52) | **0.023** |  | 0.26 (0.02 ~ 0.50) | **0.033** |
| 3 | 0.46 (0.18 ~ 0.74) | **0.001** |  | 0.16 (-0.09 ~ 0.40) | 0.203 |  | 0.17 (-0.08 ~ 0.41) | 0.180 |
| 4 | 0.63 (0.36 ~ 0.91) | **<.001** |  | 0.15 (-0.09 ~ 0.40) | 0.221 |  | 0.19 (-0.06 ~ 0.44) | 0.128 |
| CI: Confidence Interval | | | | | | | | |
| Model1: Crude | | | | | | | | |
| Model2: Adjust: gender, rural, age, education, BMI | | | | | | | | |
| Model3: Adjust: gender, marriage status, rural, Depression, sleep, age, education, hypertension, diabetes, BMI, smoking, drinking, stroke, lipid medications, coronary heart diseases | | | | | | | | |

**Table.S4** Robust regression between TC/HDL-C and total cognition scores

| Variables | β | S.E | t | β (95%CI) | *P* |
| --- | --- | --- | --- | --- | --- |
| TC/HDL quantile |  |  |  |  |  |
| 1 |  |  |  | 0.00 (Reference) |  |
| 2 | 0.37 | 0.14 | 2.69 | 0.37 (0.10 ~ 0.64) | 0.013 |
| 3 | 0.22 | 0.14 | 1.58 | 0.22 (-0.05 ~ 0.49) | 0.131 |
| 4 | 0.18 | 0.14 | 1.28 | 0.15 (-0.10 ~ 0.46) | 0.213 |

Adjust: gender, marriage status, rural, depression, sleep duration, age, education, hypertension, diabetes, BMI, smoking, drinking, stroke, lipid medication, coronary heart disease
